# Magnetic-flux-driven topological quantum phase transition and manipulation of perfect edge states in graphene tube

**DOI:** 10.1038/srep31953

**Published:** 2016-08-24

**Authors:** S. Lin, G. Zhang, C. Li, Z. Song

**Affiliations:** 1 School of Physics, Nankai University, Tianjin 300071, China; 2 College of Physics and Materials Science, Tianjin Normal University, Tianjin 300387, China

## Abstract

We study the tight-binding model for a graphene tube with perimeter *N* threaded by a magnetic field. We show exactly that this model has different nontrivial topological phases as the flux changes. The winding number, as an indicator of topological quantum phase transition (QPT) fixes at *N/*3 if *N/*3 equals to its integer part [*N/*3], otherwise it jumps between [*N/*3] and [*N/*3] + 1 periodically as the flux varies a flux quantum. For an open tube with zigzag boundary condition, exact edge states are obtained. There exist two perfect midgap edge states, in which the particle is completely located at the boundary, even for a tube with finite length. The threading flux can be employed to control the quantum states: transferring the perfect edge state from one end to the other, or generating maximal entanglement between them.

As a two-dimensional carbon sheet of single-atom thickness, graphene has been used as one kind of promising material for many aspects of display screens, electric circuits, and solar cells, as well as various medical, chemical and industrial processes^1^,^2^,^3^,^4^,^5^,^6^. Due to its special structure, graphene possesses a lot of unique properties in chemistry, physics and mechanics^7^,^8^,^9^,^10^,^11^,^12^,^13^,^14^,^15^,^16^,^17^,^18^,^19^,^20^,^21^,^22^,^23^,^24^. Recently, 3D graphene materials have received much attention, since they not only possess the intrinsic properties of 2D graphene sheets, but also provide the advanced functions with improved performance in various applications^25^,^26^,^27^,^28^,^29^,^30^.

Between the two regimes, a geometrical tube system, as a quasi-3D graphene, is expected to exhibit novel properties, especially under the control of external degrees of freedom. In this paper, we theoretically investigate a graphene tube threaded by a magnetic field in the tight-binding framework. We show exactly that a honeycomb lattice tube can be reduced to two equivalent Hamiltonians with a tunable distortion by flux, one of which is a combination of several independent dimerized models. And the other one is a 1D system with long-range hoppings. The flux drives the QPTs with multi-critical points. Applying the geometrical representation on the two equivalent Hamiltonians, we find that, although two loops show different geometries, they always have the same topology, i.e., as the flux varies the winding number around a fixed point can be the indicator of topological QPT. We also investigate the zero modes for an open tube with zigzag boundary condition by exact Bethe ansatz solutions. We find that there exist two perfect edge states at the midgap, in which the particle is completely located at the boundary, when a proper flux is applied. Remarkably, such zero modes still appear in a tube with small length, which allows us to design a nanoscale quantum device. Using the threading flux as an external control parameter, the perfect edge state can be transferred from one end to the other, or the maximal entanglement between them can be generated by an adiabatic process.

## Results

### Model and solutions

**Figure 1:**
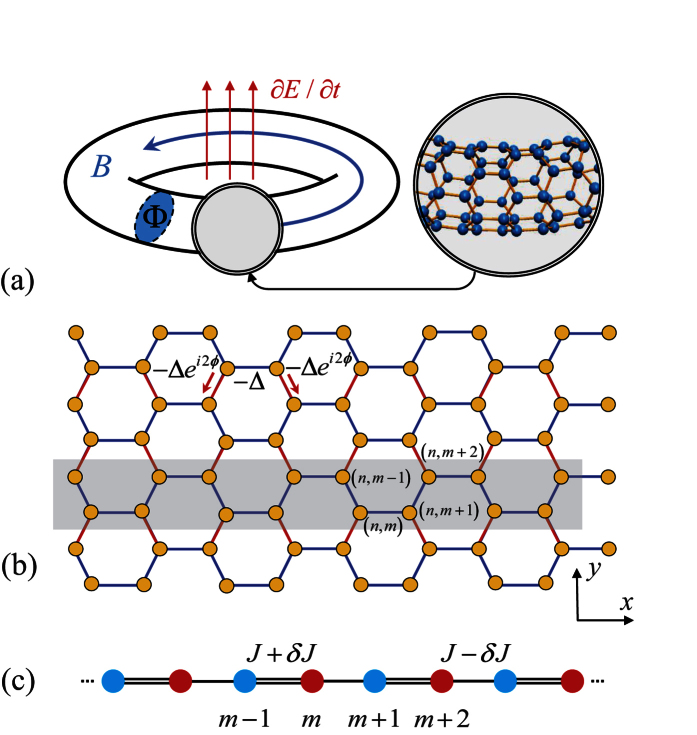
(**a**) Sketch of a graphene tube threaded by a magnetic field considered in this work. The flux induced by an external varying electric field or current, derives the topological QPTs and quantum state transfer. (**b**) Honeycomb lattice of a tube with periodic boundary condition. The tube shows translational symmetry along *x* and *y* axes. The complex hopping constants induced by the flux are denoted in red. The gray shaded region represents a lattice ring as the super unit cell of the tube, which repeats itself along *y* axis. (**c**) Sketch of a SSH lattice ring reduced from the tube. The hopping integral *J* and dimerization strength *δ* are the functions of flux.

The tight-binding model for a honeycomb tube in the presence of a threading magnetic field can be described by the Hamiltonian


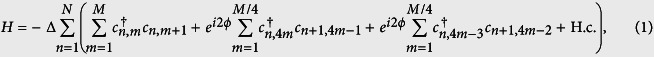


where *c*_*n*,*m*_ (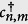) annihilates (creates) an electron at site (*n*, *m*) on an *N* × *M* lattice with integer *M*/4 and 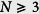, and obeys the periodic boundary conditions, *c*_*n*,*M*+1_ = *c*_*n*,1_, *c*_*N*+1,4*m*−1_ = *c*_1,4*m*−1_, and *c*_*N*+1,4*m*−2_ = *c*_1,4*m*−2_, with *n* ∈ [1, *N*], *m* ∈ [1, *M*/4]. Parameter Δ is the hopping integral with zero magnetic field (we take Δ = 1 hereafter for simplification) and *ϕ* = *π*Φ/(*N*Φ_0_), where Φ is the flux threading the tube, Φ_0_ is flux quantum. In Fig. 1, the geometry of the model is illustrated schematically. We are interested in the effect of *ϕ* on the property of the system with small *N* and large *M*. To this end, a proper form of solution is crucial. We employ the Fourier transformation 
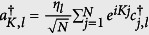
, with


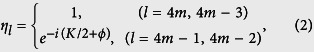


to rewrite the Hamiltonian, where *m* ∈ [1, *M*/4], and *K* = 2*πn*/*N*, *n* ∈ [1, *N*]. The Hamiltonian can be expressed as 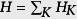, where


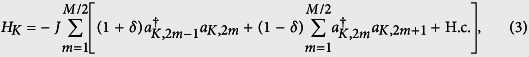


with periodic boundary *a*_*K*,*M*+1_ = *a*_*K*,1_. Together with [*H*_*K*_, *H*_*K*′_] = 0, we find that *H* is a combination of *N* independent Peierls rings with the (*K*, *ϕ*)-dependent hopping integral *J* (*K*, *ϕ*) and dimerization strength *δ* (*K*, *ϕ*), where *δ* = 1 − 1/*J* and *J* = cos(*K*/2 + *ϕ*) + 1/2. The one-dimensional dimerized Peierls system at half-filling, proposed by Su, Schrieffer, and Heeger (SSH) to model polyacetylene^31^,^32^, is the prototype of a topologically nontrivial band insulator with a symmetry protected topological phase^33^,^34^. In recent years, it has been attracted much attention and extensive studies have been demonstrated^35^,^36^,^37^,^38^,^39^. For simplicity, *H*_*K*_ can be expressed as


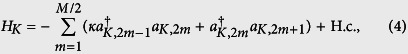


by defining *κ* = *J*(1 + *δ*). The topologically nontrivial phase for the Hamiltonian *H*_*K*_ with even *M* is realized for |*κ*| < 1. It can only be transformed into a topological trivial phase by either breaking the symmetries which protect it or by closing the excitation gap. We note that *κ* is the function of *ϕ*, which indicates the flux can drive a topological QPT. The present SSH ring described by *H*_*K*_ could lead to two transition points at *κ* =  ± 1, respectively, which will be seen from another point of view in the next section.

### Topological characterizations

**Figure 2:**
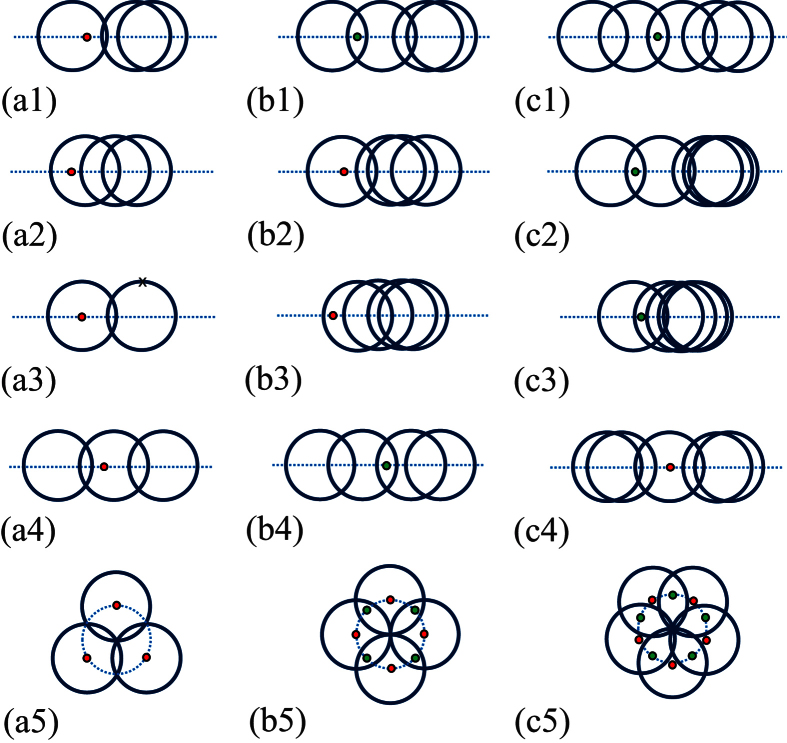
Several typical graphs in the parameter space for the graphene tubes with *N* = 3, 4, and 5. The graphs are *N* circles with unit radius. The flux determines the positions of the circle centers. The red and green circles indicate the positions of the origin. The corresponding winding numbers are [*N*/3] or [*N*/3] + 1, when the origin circles are red or green. (a1–4), (b1–4), and (c1–4) are obtained by the first equivalent Hamiltonian in Eq. (1), while (a5), (b5), and (c5) by the second equivalent Hamiltonian in Eq. (18). The cross in (a2) indicates two superposed circles.

Now we investigate the QPT from another perspective. For each SSH ring, we can represent *H*_*K*_ in a simple form


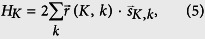


where 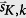 is defined as pseudo spin operators 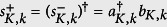 and 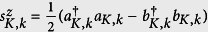, which obey the Lie algebra relations 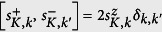 and 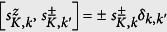. Here the fermion operators in *k* space are 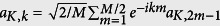 and 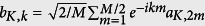 with *k* = 4*πn*/*M*, *n* ∈ [1, *M*/2], and the components of the field 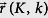 in the rectangular coordinates are


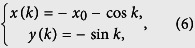


with *x*_0_ = *κ*. According to the analysis in ref. 40, the quantum phase has a connection to the geometry of the curves with parameter equation {*x*(*k*), *y*(*k*)}. Specifically, the phase diagram can be characterized by the winding number of the loop around the origin. We note that the loop of Eq. (6) represents a circle with unit radius and the center of the circle is (*K*, *ϕ*)-dependent, i.e., *x*_0_ = 2 cos(*K*/2 + *ϕ*). For any given *K*, as the flux *ϕ* changes, *x*_0_ varies within the region [−2, 2]. When *x*_0_ = ±1, the circle crosses the origin, leading to the switch of the winding number of the loops around the origin. Since a given honeycomb tube consists of a set of SSH rings, the topology of the corresponding circles reflects the feature of the system. A straightforward analysis shows that the winding number *ν* depends on *N* and flux *ϕ* in a simple form:


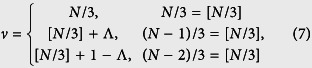


where [*x*] stands for the integer part of *x* and 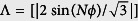. It indicates that the topology of the tube state is unchanged if *N* is a multiple of 3, or jumps between two cases, as *ϕ* varies. In Fig. 2, we demonstrate the relationship between *ν* and the geometry of the loops for several typical *N*. We have seen that the quantum phases can be characterized by the topology of the loops in the parameter space. It seems that the obtained result is based on the Fourier transformation. However, the theory of topological insulator claims that the topological character is independent of the representation and can be observed in experiment. To demonstrate this point, we consider another representation, which maps the original Hamiltonian to a single ring but with long-range hoppings and is shown in detail in Methods section. The geometrical representation is clear, still consisting of *N* cycles with unit radius. The centers of these circles locate at another unit-radius circle with equal distance, called circle-center circle. As flux varies, the origin of the parameter space moves along the circle-center circle, resulting in various values of winding numbers. A straightforward analysis shows that although the pattern is different, it presents the same topology with the first representation and Fig. 2 illustrates the patterns for small *N*.

### Control of perfect edge states

The above results indicate that the quantum phase of the model *H* exhibits topological characterization. Another way to unveil the hidden topology behind the model is exploring the zero modes of the system with open boundary condition. Consider the graphene system with zigzag boundary and its Hamiltonian could be rewritten as


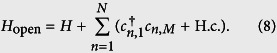


**Figure 3:**
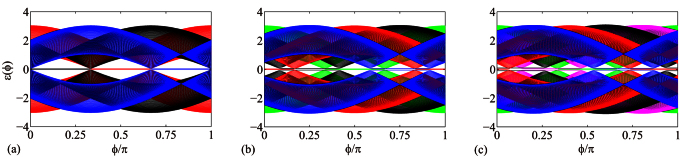
Energy spectrum of the finite-sized graphene tube (**a**) *N* = 3, *M* = 124, (**b**) *N* = 4, *M* = 124, and (**c**) *N* = 5, *M* = 124, with flux *ϕ*. In order to present the zero modes clearly, we separate the upper and lower bands by shifting them up and down with a small amount of energy, respectively. It shows that the zero modes appear and disappear periodically as the flux changes. Here the energy spectrum *ε*(*ϕ*) is expressed in units of the hopping constant Δ.

Performing the Fourier transformation, *H*_open_ can be decomposed into *N* independent SSH chains. The number of zero modes is determined by the sign of |*κ*| − 1, which leads to the same conclusion as that from the above two geometrical representations. In Fig. 3 we plots the band structures for the systems demonstrated in Fig. 2. It clearly demonstrates the processes of the emergence and disappearance of zero modes. According to the Bethe ansaz results (see Methods section.), the exact wave functions of edge states for a finite *N* but infinite *M* tube can be expressed as


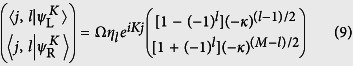


where 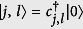 denotes the position state and 
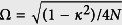
. Here 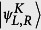 represents the edge state at left or right. The features of the edge states are obvious: (i) Nonzero probability is only located at the same one triangular sublattice. Then they have no chirality, without current for any flux, which is different from the square lattice^41^,^42^,^43^. (ii) In the case of *κ* = 0, i.e., cos(*K*_c_/2 + *ϕ*_c_) = 0, 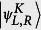 is reduced to the perfect edge state


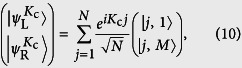


even for finite *M*, in which the particle is completely located at the boundary. Perfect edge states 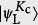 and 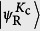 can be regarded as the qubit states 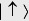 and 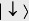 of a nanoscale qubit protected by the gap or topology. The qubit states can be controlled by varying the flux adiabatically. We take 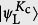 as an initial state, for example. As flux changes adiabatically from *ϕ*_c_, state 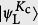 is separated as two instantaneous eigenstates at the edges of two bands. When *ϕ* (*τ*) = *ϕ*_c_ + *nπ* with *n* = 0, 1, 2, ..., the evolved state becomes an edge state again. However, it may be the superposition of two edge states, i.e., 
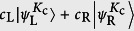
, where the coefficients *c*_L_ and *c*_R_ arise from the dynamical phase, *c*_L_ = cos*α*, *c*_R_ = *i*sin*α*, and 
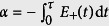
, depending on the passage of the adiabatical process. Here *E*_+_(*t*) is the eigenenergy at the edge of positive band. Proper passage allows to obtain the maximal entangled edge state 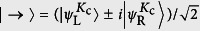, or distant edge state 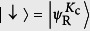.

Here we demonstrate this process by a simplest case. We consider a graphene tube with *N* = 3 and *M* = 4, which can be mapped to three 4-site SSH chains. Initially, we have *K*_c_ = 2*π*/3 and *ϕ*_c_ = *π*/6. Two perfect edge states are


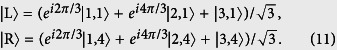


When we vary the flux adiabatically from *ϕ* (0) = *π*/6 to *ϕ* (*τ*) = 7*π*/6, state |L〉 can evolve to |R〉 if 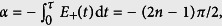 to 
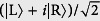
, if *α* = −(4*n* − 3)*π*/4, and to 
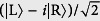
, if *α* = −(4*n* − 1)*π*/4, where *n* is a large positive integer and 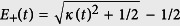. For large *N*, the Zener tunneling occurs, reducing the fidelity of quantum control schemes. In order to demonstrate the scheme, we perform the numerical simulations to compute the evolved wave function


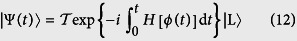


where *ϕ*(*t*) takes a Gaussian form,


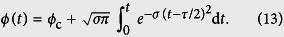


**Figure 4:**
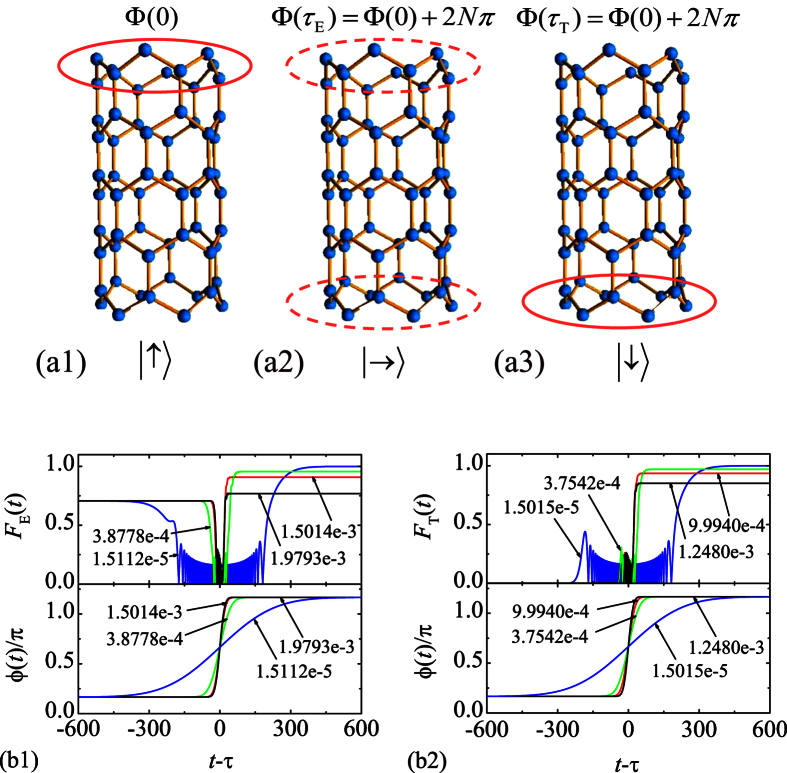
Schematic illustration of the manipulation for a perfect edge state in a graphene tube, which is represented by a red circle. When varying the flux slowly, state (a1) can evolve to (a2) and (a3), where (a2) represents a maximal entangled state. (b1) and (b2) are plots of the fidelities under the control of Gaussian pulse fluxes with several typical *σ*. Here the time *t* is in units of 1/Δ.

We employ the fidelities


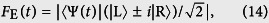



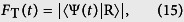


to demonstrate the two schemes. Under the assumption of adiabatical process, *F*_E_ (*t*) and *F*_T_ (*t*) reach unit when *σ* takes some discrete values, meeting the restrictions for *α* (*τ*) and *ϕ* (*τ*). However, Zener tunneling will influence the fidelity as *σ* increases. We plot the fidelities in Fig. 4 as functions of time with several typical values of *σ* for *N* = 3, *M* = 24. We see that this scheme is achievable with high fidelity even for the quasi-adiabatic process.

To conclude our analysis, we briefly comment on the experimental prospects of detecting topological phase transition, edge states, and quantum state transfer. Artificial honeycomb lattices have been designed and fabricated in semiconductors^44^,^45^,^46^, molecule-by-molecule assembly^47^, optical lattices^48^,^49^,^50^, and photonic crystals^51^,^52^,^53^,^54^. These offer a tunable platform for studying their topological and correlated phases. According to our analysis above, the topological phase transition in honeycomb lattice is equivalent to that in the SSH model. In fact, the Zak phase which is a phase degree of freedom for the SSH model is measured in reciprocal space by using spin-echo interferometry with ultracold atoms^55^. As for the detection of the perfect edge states found in our work, we propose the following scheme. We note that a perfect zero-mode state is independent of *M*, i.e., it can appear in a small sized system.

We consider a graphene tube with *N* = 4 and *M* = 4, as an example to illustrate the main point. In the absence of flux, there are two perfect edge states


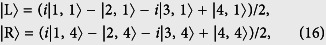


which have zero energy and are well separated from other levels. When a small flux *ϕ* is switched on, the two degenerate levels are splitted as ±4*ϕ*^2^. In the half-filled case, a small graphene tube acts as a two-level system. This artificial atom can be detected by the absorption and the emission of photons with frequency resonating to 8*ϕ*^2^.

**Figure 5:**
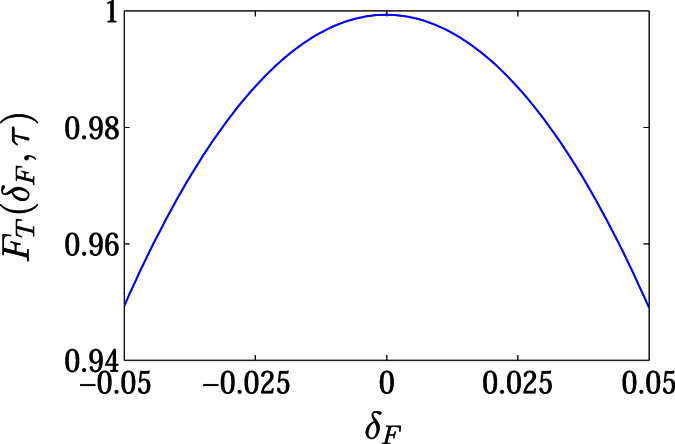
Plots of the fidelities under the control of Gaussian pulses with several typical deviations from the standard.

For the experimental realization of quantum state transfer, the varying flux affects the fidelity in the following two aspects in practice. (i) The deviation of a magnetic flux from the optimal form in the adiabatic limit. Here we consider a pulse flux as the form


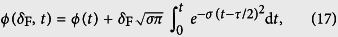


where *δ*_F_ is introduced as a quantity to express the strength of deviation. Figure 5 is the plots of the fidelities under the control of Gaussian pulse fluxes *ϕ* (*δ*_F_, *t*), which are obtained by numerical simulations for several typical values of *δ*_F_. It indicates that the deviation of the flux would reduce the fidelity for the process of adiabatic transfer. And we believe that a similar conclusion could also be achieved from the process of generating the maximal entanglement. (ii) The speed of varying flux. The adiabatic process requires a sufficiently slow speed of the flux. A fast pulse would induce to Zener tunneling, reducing the fidelity. From Fig. 4, we can see the influence of the speed on the fidelity, which provides a theoretical estimation for experiments.

## Discussion

We have proposed two ways to rewrite the Hamiltonian of a flux-threaded graphene tube by the sum of several independent sub-Hamiltonians. Each of them represents a system that may have nontrivial topology or not, which can be determined by the threading flux. For an open tube, such topology emerges as zero-mode edge states according to the bulk-boundary correspondence, which is still controllable by the flux. In addition, we have shown the existence of the perfect edge state even for a small length tube. Such kind of states can be transferred and entangled by adiabatically varying the flux, which has the potential application to design a nanoscale quantum device. There are three advantages as a quantum device: (i) The perfect edge states of two ends are well distinguishable, pointer states, even for a small length tube, (ii) Midgap states are well protected by the gap, (iii) Owing to the finite size effect, the gap between the controlled levels and others is finite for any flux, suppressing the Zener tunneling and allowing the realization of an adiabatic process.

## Methods

### Second equivalent Hamiltonian

In this section, we investigate the topological quantum phase transition in a graphene tube and propose an implementation on the transfer and entanglement of perfect edge states driven by a threading magnetic field. The notes here provide additional details on the derivations of an alternative geometrical representation of the system with periodic boundary condition and the wave functions of edge states for the system with open boundary condition. A demonstration of perfect edge states and the processes of state transfer and entanglement generation is presented.

**Figure 6:**
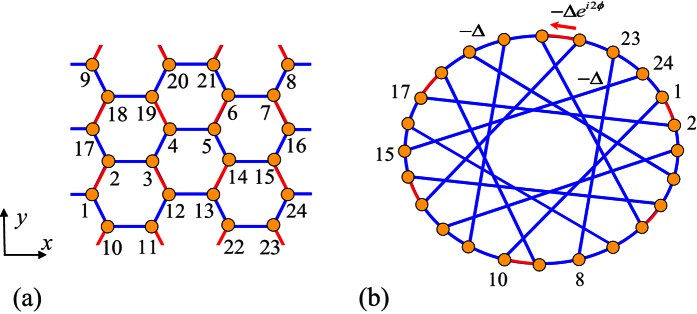
(**a**) Sketch of a graphene tube with periodic boundary condition for *N* = 3, *M* = 8. The lattice is indexed in another way. (**b**) Based on this method, the original lattice can be regarded as an 24-site ring with long-range hoppings.

In order to demonstrate the obtained winding numbers in Eq. (7) are topological invariant, we consider another way to exhibit the topological feature of the honeycomb tube. We index the lattice in an alternative way, which is illustrated in Fig. 6 for the case with *N* = 3, *M* = 8. Now we consider an *N*_0_-site tube lattice with *N*_0_ = *MN*, where *M* and *N* satisfy the condition *C* × *N* + 1 = *D* × *B*. Here *B* = *M*/4 − *N*[*M*/(4*N*)], *C* and *D* are the minimum non-negative integers that meet the condition. For example, taking *N* = 3, *M* can be taken as 12*m* − 4 (*B* = 2, *C* = 1, *D* = 2, *m* ≥ 1) while taking *N* = 4, *M* can be taken as 16*m* − 12 (*B* = 1, *C* = 0, *D* = 1, *m* ≥ 1). For finite *N*, *M* can be infinite, leading to a thermodynamic limit system, which shares the same property as that mentioned in the former sections. Such an arrangement allows us to rewrite the Hamiltonian in the form


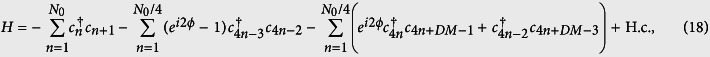


with the periodic boundary condition 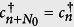. It represents an *N*_0_-site ring with long-range hoppings. Figure 6 schematically illustrates the lattice structure. Being the same system, we want to examine what topology is hidden in the new Hamiltonian, although we believe that it should be invariant. To this end, we introduce alternative Fourier transformations


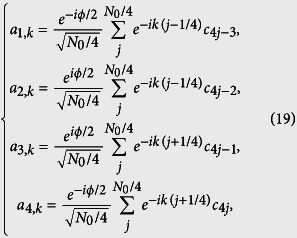


where *k* = 8*πl*/*N*_0_, (*l* ∈ 1, *N*_0_/4]). Since the translational symmetry of the Hamiltonian (18), we still have 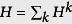 with [*H*^*k*^, *H*^*k*′^] = 0, where


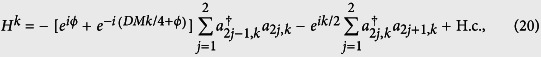


with the periodic boundary *a*_5,*k*_ = *a*_1,*k*_. Similarly, we have the corresponding pseudo-spin representation


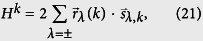


where 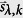 (*λ* = ±) is defined as pseudo spin operators


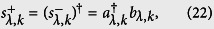



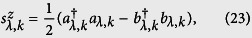


which obey


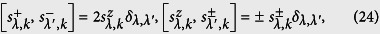


and here *a*_*λ*,*k*_ and *b*_*λ*,*k*_ are defined as


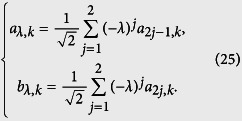


The field 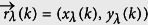 is


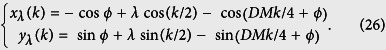


We are interested in the geometry of the curve represented by the above parameter equations. We note that


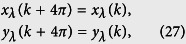


and


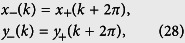


which allow us to rewrite the Hamiltonian as


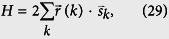


where the field and pseudo-spin operator are redefined as


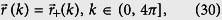



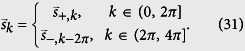


The equivalent Hamiltonian (18) provides another platform to study the geometry of the band. We find that 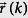 is not smooth as *k* increases continuously from 0 to 4*π*, which indicates that the curve 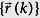 consists of several independent loops. Actually, considering discrete *k*, we can decompose the set of *k* into *N* groups by dividing *l* into *l*_*n*_ = *mN* + *n* with *m* ∈ [0, *M* − 1], *n* ∈ [1, *N*]. Then we have


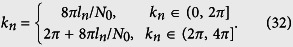


Noting *DMk*_*n*_/4 = 2*πD*(*m* + *n*/*N*), the parameter equation of the *n*th curve becomes


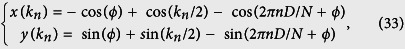


which could be rewritten as


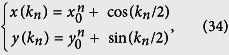


where


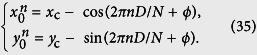


with


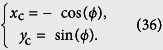


The geometry of the curves are obvious, representing *N* circles of unit radius with the center 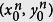. Furthermore, the positions of circle center are simply characterized as 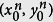. These circle centers would contribute a *N* regular polygon and be concyclic points of a circumcircle with the circumcenter (*x*_c_, *y*_c_) and the unit circumradius.

### Bethe ansatz of Edge states

Now we turn to the derivation of the wave functions of the edge states for infinite-*M* system. In the single-particle invariant subspace, the Hamiltonian of Eq. (4) with open boundary is written as


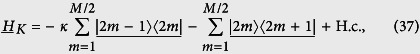


where 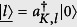 is a position state on the SSH chain. We are interested in the edge state, which corresponds to the bound state at the ends for infinite *M*. We focus on the bound state at the end of small *m*, then the other one with largest *m* is straightforward.

The Bethe ansatz wave function has the form


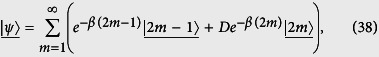


and the Schrodinger equation 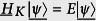 gives


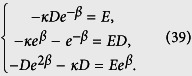


Solving these equations, we have


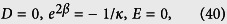


in the case of 0 < |*κ*| < 1. Then the normalized bound state wave function yields


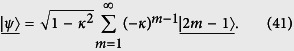


**Figure 7:**
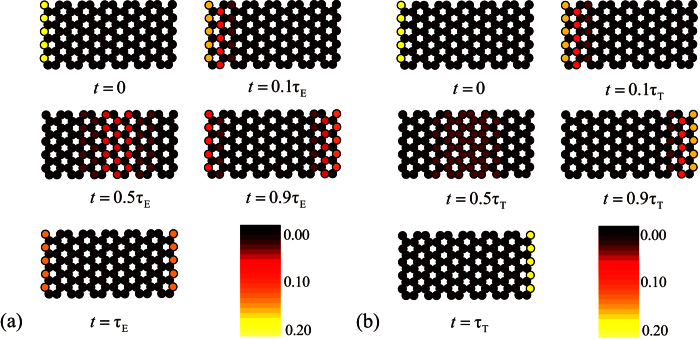
The stroboscopic picture of the probability distribution function for a perfect edge state and the dynamic process as flux varies in the graphene tube with *N* = 5, *M* = 24, and *K*_*c*_ = 4*π*/5, obtained by numerical simulations. The initial state is a perfect edge state with all probability completely distributes at the 5 leftmost sites. (**a**) Generation of a maximal entangled edge state with duration time *τ*_E_ = 8.9760 × 10^3^Δ^−1^. (**b**) Transfer of edge state with *τ*_T_ = 6.2831 × 10^3^Δ^−1^.

While, in the case of *κ* = 0, from the Hamiltonian (37) it is easy to check that there exist a perfect edge state 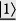 and two types of highly degenerate eigenstates 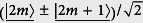 with their eigenenergies ∓1 (here *m* ≥ 1). To demonstrate the processes of perfect transfer and entanglement generation for a perfect edge state, we plot the profile of probability distributions at several typical time in Fig. 7. The results are obtained by exact diagonalization.

## Additional Information

**How to cite this article**: Lin, S. *et al.* Magnetic-flux-driven topological quantum phase transition and manipulation of perfect edge states in graphene tube. *Sci. Rep.* **6**, 31953; doi: 10.1038/srep31953 (2016).
